# Potential Impact of Benzodiazepine Use on the Rate of Hip Fractures in Five Large European Countries and the United States

**DOI:** 10.1007/s00223-012-9603-8

**Published:** 2012-05-08

**Authors:** T. P. Khong, F. de Vries, J. S. B. Goldenberg, O. H. Klungel, N. J. Robinson, Luisa Ibáñez, H. Petri

**Affiliations:** 1 Division of Pharmacoepidemiology and Clinical Pharmacology, Utrecht Institute for Pharmaceutical Sciences (UIPS), Utrecht University, Universiteitsweg 99, Utrecht, The Netherlands; 2 Epidemiology and Patient Reported Outcomes, Roche Products Limited, Shire Park, 6 Falcon Way, Welwyn Garden City, AL7 1TW UK; 3 MRC Lifecourse Epidemiology Unit, University of Southampton, Southampton, UK; 4 Department of Clinical Pharmacy and Toxicology, Maastricht University Medical Centre+, Maastricht, The Netherlands; 5 Epidemiology and Patient Reported Outcomes, Hoffman-La Roche, Building 663, Grenzacherstrasse 124, 4070 Basel, Switzerland; 6 Department of Pharmacology, Therapeutics and Toxicology, Foundation of the Catalan Institute of Pharmacology (FICF), Autonomous University of Barcelona, Barcelona, Spain; 7 Clinical Pharmacology Service, Vall d’Hebron University Hospital, Barcelona, Spain

**Keywords:** Hip fracture, Benzodiazepine, Drug utilization, Pharmacoepidemiology, Relative risk, Population attributable risk

## Abstract

Benzodiazepine use increases the risk of falls and has been associated with an increased risk of hip fractures. Our aim was to estimate the possible population impact of the use of benzodiazepines on the rate of hip fracture in France, Germany, Italy, Spain, the United Kingdom, and the United States. We conducted a literature review to estimate the pooled relative risk (RR) for hip fractures and use of benzodiazepines. Prevalence rates of benzodiazepine use in 2009 were calculated for each country using the IMS MIDAS database and three public databases in Denmark, the Netherlands, and Norway. Both the RR and prevalence rates were used for calculation of population attributable risks (PARs) of hip fractures associated with benzodiazepine use*.* The literature review showed an increased risk of hip fractures in benzodiazepine users (RR = 1.4, 95 % CI 1.2–1.6). Rate of benzodiazepine use showed considerable differences between countries, ranging from 4.7 % to 22.3 % of population ever in a 1-year period. These are reflected in results for the PARs; estimated attributions of benzodiazepines to the rate of hip fractures were 1.8 %, 95 % CI 1.1–2.6 (Germany); 2.0 %, 95 % CI 1.2–2.8 (United Kingdom); 5.2 %, 95 % CI 3.2–7.3 (Italy); 7.4 %, 95 % CI 4.5–10.0 (France); 8.0 %, 95 % CI 4.9–11.0 (United States); and 8.2 %, 95 % CI 5.1–12.0 (Spain). PAR estimates suggest that the potential attribution of benzodiazepine use on the population rate of hip fractures in the five specified European countries and the United States varies between 1.8 % and 8.2 %. During the next phase of the IMI-PROTECT study, a comparison with individual patient data will show whether this approach is valid.

In 1990, there was an estimated total of 1.26 million hip fractures worldwide. This number is expected to increase to 2.6 million in 2025 and 4.5 million in 2050, mainly as a consequence of population aging [1]. Hip fractures mainly affect the elderly, especially women [2, 3]. In general, hip fractures have significant consequences on individuals and health-care systems [2]. They are an important source of morbidity and mortality: 20–30 % of hip fracture patients die within 1 year after the fracture, and one-third are totally dependent or reside in a nursing home after 1 year [4, 5]. Hip fractures can also cause high managements costs; costs of one hip fracture are estimated at US$21,000 in the first year after surgery [2, 5, 6].

Osteoporosis is a common condition in the elderly, and hip fractures are considered the most serious consequence of osteoporosis [7]. The vast majority (90–95 %) of all hip fractures result from falling [8]. Over the past 25 years, epidemiological studies have reported a positive association between falling, hip fractures, and use of benzodiazepines, due to their sedative and muscle-relaxant effects [9, 10]. For example, a recent meta-analysis [11] showed that risk of falling was 1.5-fold increased in users of benzodiazepines.

There are no recently published studies comparing consumption of benzodiazepines across multiple countries and estimating their possible population impact on hip fractures. Drug-consumption data at the patient level are generally not publicly available in the largest European Union (EU) countries and the United States. For this study we used volume sales data of the Intercontinental Medical Statistics (IMS) database. The IMS collects drug-utilization data worldwide and attempts to do this in a standardized way. Access to these data is subject to contract. This study is part of the IMI PROTECT program, which is “a collaborative European project that comprises a programme to address limitations of current methods in the field of pharmacoepidemiology and pharmacovigilance” [12]. Our study explores the suitability of IMS data for pharmacoepidemiological studies. Specifically, its aim was to estimate the possible population impact of the use of benzodiazepines on the rate of hip fracture in five large European countries (France, Germany, Italy, Spain, and the United Kingdom) and the United States.

## Methods

In order to estimate the population impact of benzodiazepine use on the rate of hip fracture in various countries, we calculated country-specific prevalence rates of benzodiazepine use. In addition, a review was conducted to obtain pooled relative risks (RRs) of the association between benzodiazepine use and hip fractures. These were then combined into a population attributable risk (PAR) using the following formula:

1 $$ \, \rm PAR\% =  \frac{{\rm Pe  \left( {\rm RR - 1} \right)}}{{1
+ \rm Pe  \left( {\rm RR - 1} \right)}}*100 \, $$

where* Pe* is prevalence of benzodiazepine use [13].

### Literature Review

Databases PubMed, the Cochrane Library, and Embase were systematically searched in September and October 2010 with terms that related to hip fractures and benzodiazepines. Unpublished studies were not considered.

### Inclusion Criteria

Studies were included if the following criteria were reached: (1) they were reviews or observational (prospective or retrospective) and community-based (hospital-based studies were excluded as they were considered to have a weaker design because of the difficulty of finding appropriate controls [14]), (2) outcome of interest was hip fracture, (3) exposure of interest was current use of (long-acting and/or short-acting) benzodiazepines, (4) they showed RRs or odds ratios (ORs) and 95 % confidence intervals (CIs), and (5) they were published in English. During the first literature search, we included only observational studies and reviews that were published after January 1, 2000. During the second literature search, we included observational studies from the literature reviews, regardless of their publication date. However, these observational studies had to comply with the inclusion criteria.

### Data Analysis

Cochrane Review Manager (version 5, http://www.cochrane.org/) was used to calculate a pooled RR and its 95 % CI for each category under the assumption of a random-effects model. We assumed that a hip fracture is a rare disease and that the OR is an approximation of the RR. Pooled RRs were estimated for different exposure categories; any benzodiazepine, short-acting benzodiazepines (SABs) with elimination half-life (*t*_½_) <24 hours, and long-acting benzodiazepines (LABs) with *t*_½_ ≥24 hours.

### Database Studies

#### Source Populations 

In order to make projections of benzodiazepine use on the country-specific PARs of hip fracture, available benzodiazepine-sales data from the five large EU countries France, Germany, Italy, Spain, and the United Kingdom (2009); the United States (2009); Denmark (2007); the Netherlands (2008); and Norway (2008) were retrieved from the IMS database. Data from the Netherlands were not the most recent data because the Dutch 2009 reimbursement data did not match IMS sales data of 2009. This was a consequence of new benzodiazepine-reimbursement regulations in January 2009 [15].

For our study we used the IMS Multinational Integrated Data Analysis System (MIDAS) database, which is a commercial database that contains data on a global level. In some countries, IMS collects data only from pharmacies. However, in most countries, data are collected from sales from wholesalers to retail or hospital pharmacies and sales from manufacturers to retail or hospital pharmacies. The IMS uses a sample of a number of retail or hospital pharmacies and wholesalers and projects this to estimate sales for all retail and hospital pharmacies in a country. In the IMS MIDAS database, data are registered per drug and for all its application forms. These drugs are categorized according to the Anatomical Classification of Pharmaceutical Products of the European Pharmaceutical Marketing Research Association (EphMRA) [16]. The IMS MIDAS database contains only sales data in product volume, which we converted to the World Health Organization’s (WHO’s) defined daily dosages (DDDs). In order to estimate the number of benzodiazepine users in IMS MIDAS, we used publicly available sources of three countries (Denmark, the Netherlands, Norway) that contained total numbers of DDDs as well as numbers of users of a drug. We used data sources from these three countries because equivalent types of databases from the five big EU countries and the United States were missing. All three were online databases containing benzodiazepine prescription data: the Register of Medicinal Product Statistics of the Danish Medicines Agency [17], the Dutch Genees-en hulpmiddelen Informatie Project (GIP) databank [18], and the Norwegian Prescription Database (NorPD) [19]. The database of the Danish Medicines Agency contains information, derived from Danish pharmacies, on prescribed, reimbursed drugs and over-the-counter drugs [20]. The GIP databank was set up by the Dutch Health Care Insurance Board and contains nonhospital data about prescribed, dispensed, and reimbursed drugs [21]. The NorPD database was developed by the Norwegian Institute of Public Health; it receives data on all prescribed (reimbursed or not) and dispensed drugs to all individual patients in every Norwegian pharmacy [22].

#### Exposure: Medication Selected 

In each IMS data source (i.e., for each country), we retrieved all data on benzodiazepine use. Data on benzodiazepines included in WHO ATC classes N05BA (benzodiazepine derivates), N05CD (benzodiazepine derivates), and N05CF (benzodiazepine-related drugs) were analyzed [23]. Benzodiazepines with antiepileptic effects (ATC class N03AE) were excluded because fracture risk is already increased in epilepsy and this could have introduced bias [24]. The selected benzodiazepines were then subdivided into two groups: short-acting and long-acting. This was done according to their elimination half-life in the Micromedex [25].

#### Outcome Definition 

One-year prevalence (YPr) was defined as the number of ever-users of benzodiazepines in a given calendar year divided by the total population in that same calendar year. These denominator population numbers were based on data from Eurostat and the US Census Bureau [26, 27]. A user was anyone who has had one or more prescriptions in 1 year. To estimate the number of users in each country, first IMS MIDAS sales data were converted into a number of units of the WHO’s DDD. The WHO’s definition of a DDD is “the average maintenance dose of a drug when used on its major indication in adults” [28]. Then, this total number of DDDs was used to calculate consumption of DDDs per 1,000 denominator population per day (DDD/1,000 persons/day). Expression of drug utilization in DDD/1,000 persons/day allows aggregation of data that differ in administration form and strength of dose and makes it possible to compare drug use between countries [28, 29].

Equation 2 shows a summary of the steps that were taken to convert these DDDs/1,000 persons/day to country-specific prevalence rates. For this estimation, we assumed that the prevalence was proportional to DDD/1,000 persons/day and that the ratio of mean (DDD/1,000 persons/day)_public databases_ to mean (DDD/1,000 persons/day)_IMS databases_ was equal in each country. We called this ratio the “conversion factor” and, setting the Danish, Dutch, and Norwegian databases as a standard, multiplied all IMS data by this factor.

2 $$ {\text{Prevalence rate of benzodiazepine use}} =
\frac{{{\text{\it A }} \times {\text{\it B}}}}{\text{\it C}} $$

where *A* is country-specific benzodiazepine consumption in DDD/1,000 persons/day (IMS databases, converted with conversion factor); *B* is mean prevalence of benzodiazepine use in Denmark, the Netherlands, and Norway (Danish registries, Dutch GIP databank, Norwegian NorPD database); and *C* is mean benzodiazepine consumption in DDD/1,000 persons/day in Denmark, the Netherlands, and Norway (Danish registries, GIP databank, NorPD database).

### Analysis (Combination of Literature Review and Database Studies)

The primary outcome of this study was the PAR. This is a measure that estimates how many hip fractures could be prevented if exposure to the risk factor, in this case benzodiazepine use, was eliminated [30]. PARs for each country were estimated using the pooled RR and prevalence (equation 1). This was done for each benzodiazepine category (any benzodiazepine, SABs, and LABs).

We also conducted a sensitivity analysis by calculating PARs for the whole population and for men and women 65 years and older in Denmark and Norway. YPr values were calculated using numbers of DDDs and numbers of users in the Danish and Norwegian databases and population data from Eurostat.

## Results

We identified 11 studies that met the inclusion criteria and were included for the calculation of pooled RRs for hip fractures and benzodiazepines. Five of these studies were published before January 1, 2000, but were included in review studies that were published after this date. Therefore, they also contributed to our study.

**Fig. 1:**
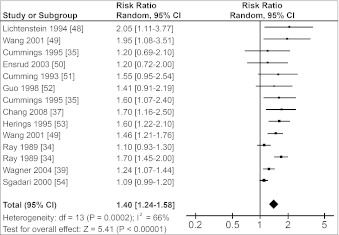
Forest plot of relative risks for hip fractures and use of benzodiazepines versus nonuse. *Squares* represent the relative risk in each study; their sizes are proportional to their weights. *Horizontal lines* represent 95 % confidence intervals. *Black diamonds* represents the pooled relative risk (calculated with a random-effects model). Studies are ordered according to their weights

**Fig. 2:**
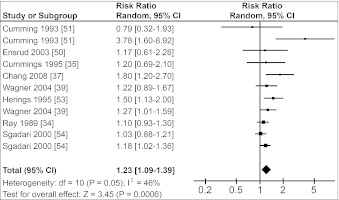
Forest plot of relative risks for hip fractures and use of short-acting benzodiazepines versus nonuse. *Squares* represent the relative risk in each study; their sizes are proportional to their weights. *Horizontal lines* represent 95 % confidence intervals. *Black diamonds* represents the pooled relative risk (calculated with a random-effects model). Studies are ordered according to their weights

**Fig. 3:**
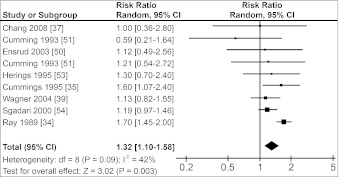
Forest plot of relative risks for hip fractures and use of long-acting benzodiazepines versus nonuse. *Squares* represent the relative risk in each study; their sizes are proportional to their weights. *Horizontal lines* represent 95 % confidence intervals. *Black diamonds* represents the pooled relative risk (calculated with a random-effects model). Studies are ordered according to their weights

Figure 1 shows that the risk of hip fractures was 1.4-fold increased in users of any benzodiazepine (RR = 1.40, 95 % CI 1.24–1.58). Pooled RRs for users of SABs and LABs were both lower: 1.23 (95 % CI 1.09–1.39) and 1.32 (95 % CI 1.10–1.58), respectively (Figs. 2, 3). There was no substantial difference between the pooled RR of cohort studies (1.32, 95 % CI 1.17–1.48) and the pooled OR of case–control studies (1.43, 95 % CI 1.20–1.71). Figures 1–3 also show the results for the heterogeneity tests. I^2^, given in percentages, quantifies how much of the variation in RRs from the included studies is a result of genuine differences between the studies rather than chance [31]. In our study, I^2^ varied between 42 % and 66 %, i.e., a moderate variation [32].

Table 1 shows that there were considerable differences in benzodiazepine use across countries. The average of benzodiazepine consumption per day was highest in Spain (85.5 DDD/1,000 persons/day) and the United States (82.9 DDD/1,000 persons/day). In each country, SABs were consumed more than LABs. As described in Methods, DDDs/1,000 persons/day, calculated with IMS data, were all multiplied by a conversion factor, which was 0.937.

**Table 1:** Benzodiazepine use (DDD/1,000 persons/day) in five European countries and the United States, calculated using IMS MIDAS drug sales data (2009)

Country	Any benzodiazepine	SAB	LAB
France	76.0	64.1	11.9
Germany	18.0	14.0	3.91
Italy	52.4	42.4	10.0
Spain	85.5	67.9	17.6
UK	19.3	11.6	7.63
US	82.9	75.9	6.96

*DDD* WHO’s defined daily dose, *SAB* short-acting benzodiazepine, *LAB* long-acting benzodiazepine

One-year prevalence rates of benzodiazepine use, calculated using the DDDs/1,000 persons/day, ranged between 4.7 % (Germany) and 22.3 % (Spain). Furthermore, prevalence rates were estimated to be between 3.0 % (Germany) and 19.7 % (United States) for SABs and 1.0 % (Germany) and 4.7 % (Spain) for LABs. These numbers were used to calculate the PARs.

Table 2 shows that the estimated attribution of use of any benzodiazepine on the risk of hip fractures varied between 1.8 % and 8.2 % and that in all countries this PAR was higher than the PARs of the two subgroups. Also, in each country, the attribution of SABs was higher than that of LABs. The PARs of any benzodiazepine and LABs were highest in Spain (respectively, 8.2 % and 1.5 %), while the PAR of SABs was highest in the United States (4.3 %).

**Table 2:** Estimated population attributable risk (%) and its 95 % confidence interval for hip fractures associated with benzodiazepine use in five European countries and the United States

Country	Any benzodiazepine^a^	SAB^a^	LAB^a^
France	7.4 (4.5–10)	3.7 (1.5–6.1)	1.0 (0.3–1.8)
Germany	1.8 (1.1–2.6)	0.8 (0.3–1.4)	0.3 (0.1–0.6)
Italy	5.2 (3.2–7.3)	2.5 (1.0–4.1)	0.8 (0.3–1.5)
Spain	8.2 (5.1–12)	3.9 (1.6–6.4)	1.5 (0.5–2.6)
UK	2.0 (1.2–2.8)	0.7 (0.3–1.2)	0.6 (0.2–1.2)
US	8.0 (4.9–11)	4.3 (1.7–7.1)	0.6 (0.2–1.1)

^a^Percentages of SABs (short-acting benzodiazepines) and LABs (long-acting benzodiazepines) cannot be combined in order to get the percentage of the total group (any benzodiazepine). See discussion for further explanation

Results of a sensitivity analysis showed that in both Denmark and Norway the PARs calculated for men and women 65 years and older were considerably higher compared to the PARs that were based on the whole population. Also, the attributable risk for hip fractures associated with benzodiazepine use was about twice as high for elderly women compared to elderly men.

## Discussion

This study found that the estimated impact of benzodiazepine use on hip fracture rate varied between 1.8 % and 8.2 % in the six countries studied (Table 2). These differences are a result of the considerable differences in benzodiazepine use between these countries; DDDs/1,000 persons/day varied from 18 to 86 (Table 1). In all countries, the PAR of SABs was higher than that of LABs. This suggests that a larger proportion of hip fractures may be associated with the use of SABs than the use of LABs.

Review of observational studies has shown that all benzodiazepines, as well as the two subgroups separately, are associated with an increased risk of hip fractures. This relationship can be explained by an increased risk of falls associated with benzodiazepine use. Previous studies reported increased falls and increased risk of hip fracture with use of LABs [33–36], while others found the same association for SABs [37–39]. Some studies found an increased risk for both benzodiazepine types, with higher risks for LABS compared to SABs [34, 35, 40]. This can be explained given that SABs have less potential for accumulation and prolonged sedative effects [34]. In contrast, Chang et al. [37] suggested that SABs may show more severe withdrawal symptoms, more rapid tolerance development, and more cognitive impairment and could therefore lead to a higher association with hip fractures than LABs.

As shown in Table 2, the PARs of SABs and LABs cannot be combined to obtain the PAR of the total group of benzodiazepines; this is a result of different studies that were used to calculate the RRs of these three categories. For calculation of the RR of “any benzodiazepine,” several extra studies were included that were not included in the calculation of the RR of SABs or LABs as results were not split by duration of action.

The heterogeneity tests in the Cochrane Review Manager (Figs. 1–3) showed that the heterogeneity (I^2^) was between 42 % and 66 %, indicating moderate heterogeneity [32]. The pooled RR for the total group of benzodiazepines showed the highest I^2^ value, probably because this group consists of studies using different types of exposure (SABs or LABs).

A strength of the IMS is that data collection is similar across countries. This allows comparison between these countries. However, the IMS MIDAS database does not contain the number of users of a drug but rather the total quantity of a drug used per country. Therefore, we estimated numbers from IMS volume data and built in a conversion factor based on the volume data and number of recipients from three public databases. A single estimate of use of benzodiazepines was used for each country, while regional variation is likely to exist. Another limitation is that we had to make certain assumptions when estimating the 1-year prevalence rate. A conversion factor, calculated from databases of three northern European countries, was used to calculate 1-year prevalence rates for other countries. Furthermore, we did not assume a change of risk from drug use over time. Literature about this so-called hazard function is limited on benzodiazepines. There was also a limitation in estimating the pooled RR; only observational studies were used because no data from clinical trials were available. These observational studies are subject to various forms of bias [41–43]. Also, all of these studies used populations 65 years and older, which could lead to an inaccurate estimation of RRs and, thus, the PARs for the whole population. Another issue is the fact that benzodiazepines are used mainly by women and the elderly [44–47]. Thus, the two groups with highest risk for hip fractures are also the groups that are most exposed to benzodiazepine use. This possibly causes an underestimation of the PARs because in this study it was assumed that benzodiazepine use is equally distributed over the whole population. The sensitivity analysis, which showed that the PAR is indeed higher for the elderly and especially for older women, confirms this.

In conclusion, in our study the estimated attribution of benzodiazepine use on the rate of hip fractures varied between 1.8 % and 8.2 %. This suggests that in each of the studied countries, a substantial number of hip fractures may be associated with the use of benzodiazepines. These numbers are different in each country, reflecting differences in consumption of these drugs. Although some assumptions were made in deriving these estimates, this study shows the possibility to use the IMS MIDAS database for country comparisons of benzodiazepine consumption. During the next phase of the IMI-PROTECT study, a comparison with individual patient data will show whether our approach can reliably estimate the impact of benzodiazepines on hip fractures in different countries. We consider databases with drug-consumption data from multiple countries to be valuable when studying similar questions with both other drugs and other outcomes.
